# Montelukast Improves Urinary Bladder Function After Complete Spinal Cord Injury in Rats

**DOI:** 10.3390/ijms26125606

**Published:** 2025-06-11

**Authors:** Elena E. Keller, Sophina Bauer, Karin Roider, Michael Kleindorfer, Peter Törzsök, Julia Tevini, Thomas Felder, Ludwig Aigner, Lukas Lusuardi

**Affiliations:** 1 Institute of Molecular and Regenerative Medicine, Paracelsus Medical University, Strubergasse 21, 5020 Salzburg, Austria; 2 Department of Urology and Andrology, University Hospital Salzburg, Müllner Hauptstrasse 48, 5020 Salzburg, Austria; 3 Faculty of Health and Sport Sciences, Széchenyi István University, 9026 Győr, Hungary; torzsok.peter@gmail.com; 4 Department of Laboratory Medicine, Paracelsus Medical University, Müllner Hauptstrasse 48, 5020 Salzburg, Austria

**Keywords:** spinal cord injury, urinary bladder, detrusor sphincter dyssynergia, inflammation, leukotriene, rat

## Abstract

Bladder dysfunction is among the most drastic and quality-of-life-reducing conditions after spinal cord injury (SCI). Neuroinflammation in the lower urinary tract (LUT) after SCI could be a key driver of neurogenic bladder dysfunction and tissue fibrosis. Leukotrienes, a group of highly active lipid mediators, are potent inflammatory mediators. Here, we explored the potential of early montelukast (MLK) therapy, a cysteinyl leukotriene receptor 1 antagonist, on LUT function and structure four weeks after severe SCI in rats. Rats (strain Lewis, female, n = 50) received a permanent bladder catheter, followed by a complete T9 spinal cord transection. MLK was given daily, starting on day one post-injury. Bladder and locomotor function were regularly assessed. Bladder tissue was histologically and immunhistochemically analyzed. Post-SCI, MLK concentrations in plasma and cerebrospinal fluid were clinically relevant. MLK improved bladder functionality. MLK had no impact on smooth muscle alignment and uroepithelial integrity at this early SCI time point. This pilot study gave first insights into early, continuous oral MLK treatment with the first promising results of preserved LUT function and possible subsequent improved tissue integrity.

## 1. Introduction

Spinal cord injury (SCI) has an annual incidence of 40 cases per million. SCI can occur after spinal cord trauma or as a consequence of vascular ischemia, infection, or others [1]. With regard to the lower urinary tract (LUT), a SCI interrupts the complex neuronal communication that is prerequisite for its proper function [2]. Lesions above the sacral micturition center (S2–S4) lead to a dyscoordination of the micturition reflex. This induces simultaneous contractions of the bladder detrusor muscle and the urethral sphincter, termed detrusor sphincter dyssynergia (DSD), and resulting high bladder pressures. These alterations of the LUT pose a risk for failure of the upper urinary tract in the long term [3,4]. Over 90% of patients with SCI suffer from multiple clinical symptoms, i.e., urinary incontinence, high post-residual volumes, and recurrent urinary tract infections, severely influencing their quality of life [5]. Current therapies, such as oral antimuscarinic drugs, adrenergic beta-3 receptors, and botulinum toxin injections, aim to reduce the various symptoms of neurogenic lower urinary tract dysfunction (nLUTD) and to protect the upper urinary tract [6].

However, drug side effects, minimal effect size, and worsening of the symptoms often lead to discontinuation of therapy. Most importantly, there is no established therapy to prevent the development of DSD and thereby caused fibrotic changes in the bladder tissue and their consequences.

Even though the central nervous system is known to be an immune-privileged site, an SCI induces a transient systemic immune-depression with an orchestrated invasion of circulating immune cells, the activation of resident microglia and astrocytes, and the expression of immune and inflammatory mediators, including complement, cytokines, and chemokines, after SCI in the context of neuroinflammation [7]. Kwiecien et al. described in detail the mechanisms of SCI pathogenesis and the close association between progressive astrogliosis and reduction in the severity of inflammation in a systematic rodent SCI study [8]. Targeting astrogliosis might be a key mechanism to generate a pro-regenerative state and alleviate functionality [9], which is also important for peripheral organs.

One of the organs that is affected by the systemic inflammatory response is the urinary bladder. So, while changes in neuronal input to the bladder play an obvious role, a major contributor to bladder dysfunction is inflammation [10].

Neurogenic inflammation after SCI in the LUT might be a key driver in the development of DSD and fibrosis. Intense research has focused on cellular mechanisms underlying neurogenic inflammation in the pelvic viscera [11]. Given the well-established inflammatory changes in the bladder and their detrimental effects, it is somewhat surprising that so few studies have examined the potential benefit of anti-inflammatory treatments [12]. One study investigated the oral administration of anti-inflammatory molecule S-nitrosoglutathione following SCI and found that it promoted the recovery of bladder function via the inhibition of inflammatory responses [13]. Similar effects were seen with simvastatin [14], dantrolene [15], and quercetin [16], all of which have anti-inflammatory properties.

Leukotrienes (LT), a group of highly active lipid mediators, are potent inflammatory mediators involved in various biological responses [2,17], including central nervous system (CNS) pathologies as an SCI [18,19]. In peripheral tissues such as the urinary bladder, leukotriene receptors are present on smooth muscle cells and on local inflammatory cells [17,20]. Inflammatory cells invading from the bloodstream into deteriorated neurogenic bladder tissue potentiate histamine hyperresponsiveness of the detrusor smooth muscle cells and mediate inflammatory bladder disorders [20,21]. The early inhibition of the leukotriene pathway with a leukotriene receptor antagonist may reduce neurogenic inflammation in the LUT after SCI, with a possible beneficial outcome on DSD. To achieve this, montelukast, a cysteinyl LT receptor 1 antagonist and FDA-approved anti-asthmatic agent [22], might be a good candidate for a repurposing drug. Montelukast has shown several beneficial effects on the urinary bladder. Oral montelukast treatment significantly decreased urinary frequency and pain in patients with interstitial cystitis [23,24]. Even more relevant, a single intraperitoneal injection of montelukast maintained bladder tissue integrity after a contusion SCI in rats [25]. With regard to neuroinflammation in the spinal cord, recent studies identified the necessity to dampen astrogliosis in order to alleviate neuroinflammation [8] and highlighted that montelukast shifted the balance of the astrocyte reactive state in the spinal cords of female mice in favor of pro-regenerative function [9].

Given montelukast’s well-established anti-inflammatory properties and long-standing clinical use, we conducted a short-term pilot study in female rats to explore its potential effect on early post-injury bladder changes following complete spinal cord transection. However, in this initial investigation, montelukast treatment was assessed in a limited manner—primarily by monitoring leukotriene levels in plasma and cerebrospinal fluid over the course of four weeks post-injury. As the pathophysiology of neurogenic lower urinary tract dysfunction (nLUTD) after SCI involves complex, time-dependent inflammatory and neuroplastic processes affecting the spinal cord and lower urinary tract, within this study, we capture an early snapshot of a multifaceted disease process.

## 2. Results

For this explorative survey, we focused on two main findings: (i) the confirmation of relevant montelukast plasma concentration in the SCI rat and (ii) the bladder-specific effects of montelukast in the SCI rat model.

### 2.1. Post-SCI Montelukast Concentrations in Plasma and Cerebrospinal Fluid (CSF) Are Clinically Relevant

**Figure 1:**
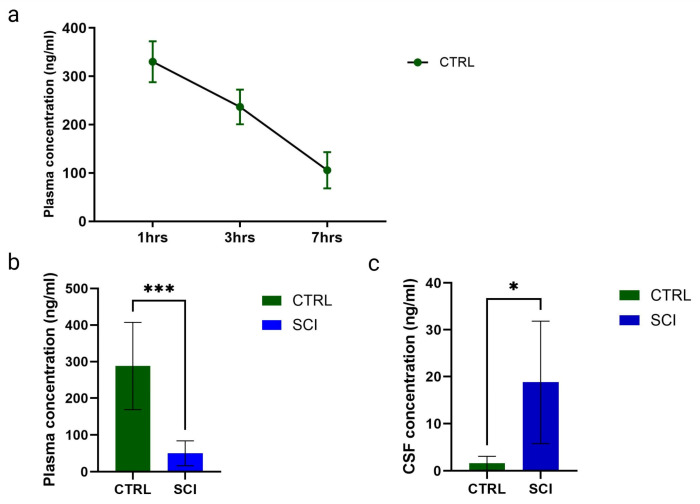
Montelukast concentrations (in ng/mL) of healthy control (CTRL) and spinal cord injured (SCI) rats. (**a**) Montelukast concentration from blood plasma of healthy controls taken at 1, 3, and 7 h (hrs) after administration. (**b**) Montelukast concentrations from blood plasma (**b**) and cerebrospinal fluid (**c**) of healthy controls and spinal cord injured rats, taken 3 h after administration. *: *p* ≤ 0.05, ***: *p* ≤ 0.001; significant differences according to unpaired *t*-test.

In healthy control animals, the montelukast plasma concentration was determined one, three, and seven hours post-administration and is shown in (Figure 1a). The montelukast plasma concentration steadily decreased over time post-uptake, with 334 ± 85 ng/mL at one hour, 169 ± 11 ng/mL at three hours, and 36 ± 17 ng/mL at seven hours.

Post-SCI, the montelukast plasma concentration was significantly lower in SCI animals, with 50.20 ng/mL (mean, SCI) versus 288.1 ng/mL (mean, healthy control), determined three hours post-administration on day post-injury 29 (Figure 1b). For the cerebrospinal fluid, the opposite was observed. In healthy control animals, the mean concentration was 1.61 ng/mL, while in SCI animals, it was significantly higher, with a mean of 18.82 ng/mL, determined three hours post-administration on day post-injury 29 (Figure 1c).

### 2.2. Montelukast Has No Influence on Locomotor Function During Four Weeks of Complete SCI

**Figure 2:**
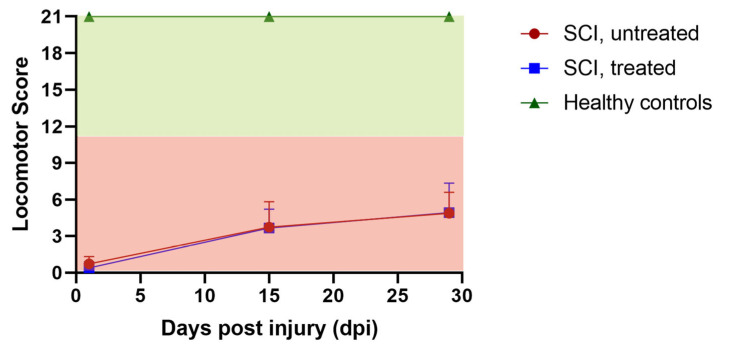
Locomotor recovery of untreated and montelukast-treated spinal cord injury (SCI) rats as well as healthy controls (CTRL) over 4 weeks. y-axis: locomotor score; x-axis: days post-injury (dpi).

The locomotor function of SCI rats was assessed at baseline and three times during SCI follow-up at 1, 15, and 29 days post-SCI, as shown in Figure 2. All baseline recordings demonstrated consistent BBB scores of 21, indicating no influence of the catheter implants and the worn infusion harnesses on locomotor function. At day 1 post-SCI, all SCI animals, irrespective of group, displayed scores between 0 and 1, indicating complete paralysis. At 29 days post-SCI, only minor motor recoveries were observed, with BBB scores ranging between 1 and 8, and with strong variations in both groups (montelukast-treated and untreated). No stepping ability was observed in any of our SCI animals, irrespective of treatment. All healthy control animals had consistent BBB scores of 21 throughout the follow-up duration. In summary, the locomotor dysfunction was highly similar in all SCI animals, irrespective of group, with only minimal signs of recovery after the complete transection of the spinal cord.

### 2.3. Montelukast Reduces Signs of DSD After a Complete SCI

**Figure 3:**
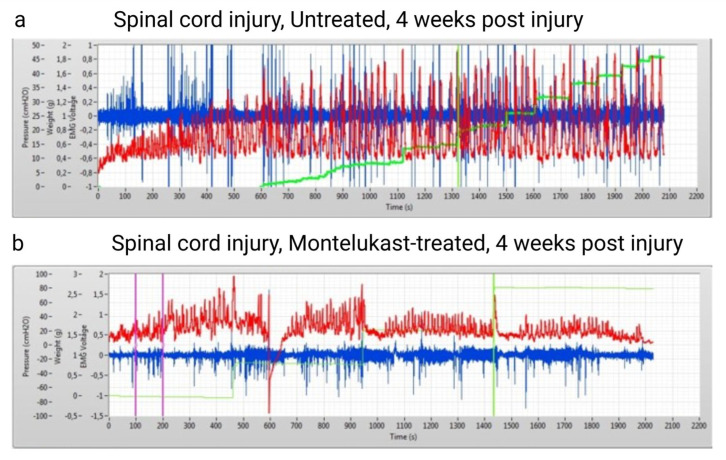
Qualitative images of two urodynamic recordings of an untreated spinal cord injury (SCI) rat (**a**) and a montelukast-treated SCI rat (**b**) 4 weeks after a spinal cord injury. Y-axis: red curve: Intravesical pressure in cmH_2_O. Green curve: voided volume (in grams). Blue: electromyogram (voltage). X axis: time in seconds.

Bladder function was assessed at baseline prior to injury and once per week starting at day 1 post-injury until 4 weeks. All rats depicted normal and regular micturitions at baseline recording. During spinal shock in week 1 post-SCI, all rats showed the typical acontractile bladders, with complete retention and urine overflow. From the second week onwards, untreated and montelukast-treated SCI rats revealed the first differences in urodynamic patterns, which developed further apart. In general, untreated SCI rats had less frequent and irregular micturitions, along with high-frequency non-voiding contractions (Figure 3a). In contrast, the montelukast-treated SCI collective showed more regular, and, at a later stage, also more frequent micturitions, which were often still accompanied by non-voiding contractions (Figure 3b). Residuals (calculated by infused volume—voided volume/per micturition cycle) were higher in untreated SCI rats.

**Figure 4:**
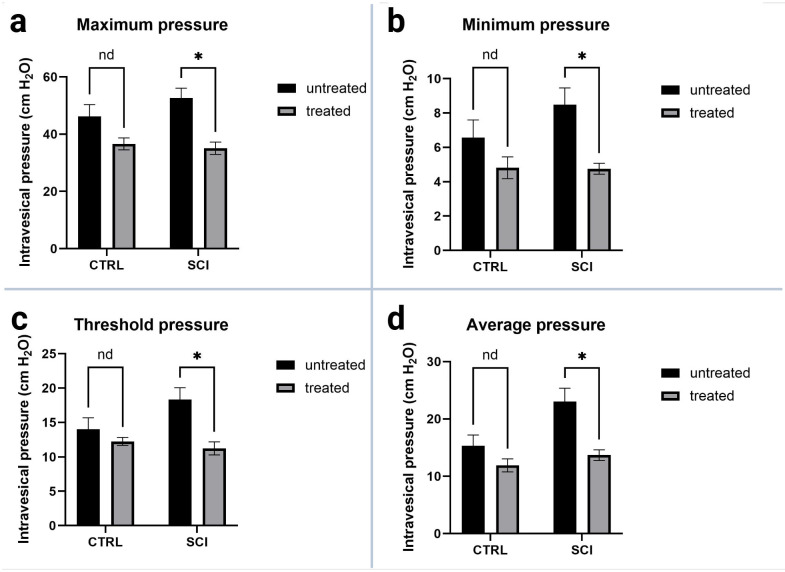
Urodynamic pressure parameters of untreated and montelukast-treated healthy (CTRL) and spinal cord injury (SCI) rats 4 weeks after SCI. (**a**): Maximum intravesical pressure; (**b**): Minimum intravesical pressure; (**c**): Threshold intravesical pressure; (**d**): Average intravesical pressure. y-axis: Intravesical pressure in cm H_2_O. x-axis: Groups. nd: not a discovery. *: *p* ≤ 0.05, significant difference. Test used: unpaired *t*-test with Welch correction.

**Figure 5:**
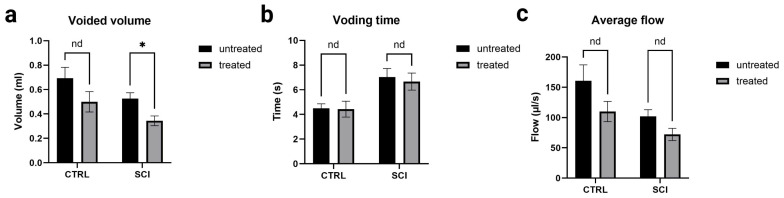
Urodynamic voiding parameters of untreated and montelukast-treated healthy (CTRL) and spinal cord injury (SCI) rats 4 weeks after SCI. y-axis (**a**): Volume in milliliters; y-axis (**b**): Time in seconds; y-axis (**c**): Flow in microliters per second. x-axis: Groups nd: not a discovery. *: significant difference. Test used: unpaired *t*-test with Welch correction.

All pressure parameters (see Figure 4) were lower in montelukast-treated animals (healthy control and SCI), with significant differences amongst the two SCI collectives with *p* = 0.000031 (maximum detrusor pressure, Figure 4a), *p* = 0.000469 (minimum voiding pressure, Figure 4b), *p* = 0.000478 (threshold pressure, Figure 4c), and *p* = 0.000362 (average pressure, Figure 4d). Regarding the voiding parameters (see Figure 5), again, all montelukast-treated animals (healthy control and SCI) depicted lower values of the parameters: voided volume, voiding time, and average flow. Among the two SCI groups, there was a significant difference in the voided volume (*p* = 0.003613; Figure 5c). No relevant differences were observed in the two other parameters. A comparative analysis of all urodynamic parameters between the montelukast-treated healthy controls and SCI groups revealed no significant differences for any of the analyzed parameters (see Table 1).

All rats showed clinical and urodynamic signs of obstruction: higher volumes at morning care, higher post-void residuals, and higher pressure in combination with lower flow.

To sum up, untreated SCI rats showed more prevalent signs of DSD with higher pressures, less regular voiding events, higher residuals, and a tendency towards neurogenic bladder outlet obstruction. On the contrary, montelukast-treated SCI rats revealed fewer signs of DSD with pressure-normalized micturition and low amounts of residuals.

### 2.4. Impact of Montelukast on Smooth Muscle Alignment and Uroepithelial Integrity Are Not Seen at This Early SCI Time Point

**Figure 6:**
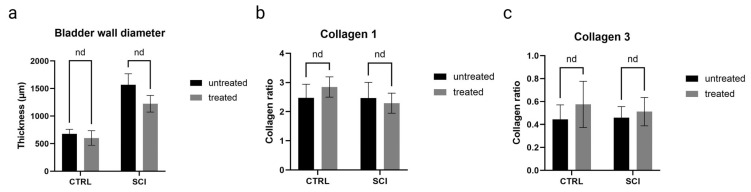
Bladder wall-specific histological analyses of untreated and montelukast-treated healthy (CTRL) and spinal cord injury (SCI) rats. (**a**) Bladder wall diameter (in micrometers); (**b**) Collagen 1 (displayed as collagen ratio); (**c**) Collagen 3 (displayed as collagen ratio. x-axis: Groups. nd: not a discovery.Test used: unpaired *t*-test with Welch correction.

**Figure 7:**
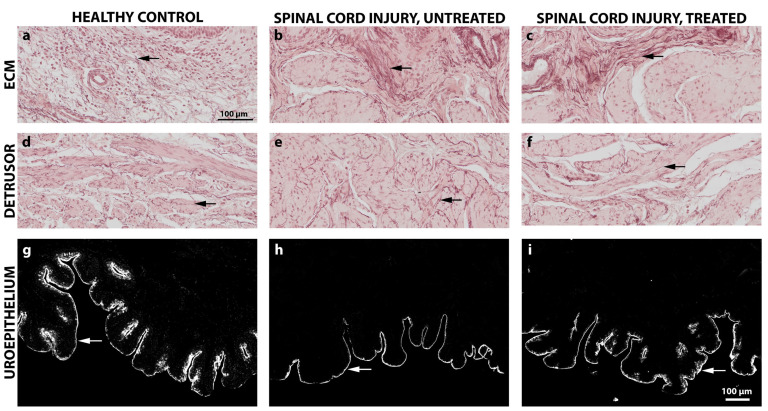
Qualitative histological and immunohistochemical images of the bladder extracellular matrix, bladder detrusor muscle, and uroepithelium of healthy control and spinal cord injured rats with or without montelukast treatment. (**a**–**f**): Orcein staining; arrows: Elastic fibers; (**g**–**i**): immunochistochemical staining of Uroplakin-III (arrow). Scale a–i: 100 µm.

The structural changes of the bladder wall were assessed 4 weeks after the onset of SCI by histological and immunohistochemical analyses. Due to the early chronic time point, only qualitative assessments of the tissue were performed. All SCI bladders, montelukast-treated or untreated, showed thickened bladder walls in contrast to the healthy control collectives (Figure 6a). Regarding the Collagen 1 and 3 ratio, montelukast-treated rats had slightly less Collagen 1 (Figure 6b) but more Collagen 3 (Figure 6c) than the untreated rats. No differences in detrusor smooth muscle alignment and suburothelial extracellular matrix composition were observed between SCI and healthy bladder tissue (Figure 7a–f). Elastic fibers appeared in both their corkscrew-like shape as well as partially stretched in the smooth muscle and in the extracellular matrix of the lamina propria (Figure 7a–f, arrows). To evaluate the integrity and barrier function of the uroepithelium, uroplakin 3 staining was utilized. All bladders, healthy and SCI, showed intact and continuous uroplakin 3-positive epithelial linings (Figure 7g–i). To summarize, no noteworthy structural differences were observed between montelukast-treated and untreated SCI bladders at this early chronic time point of 4 weeks post-SCI.

## 3. Discussion

The aim of this study was to explore the influence of montelukast, a cysteinyl leukotriene receptor 1 antagonist, on neurogenic bladder development and structural changes after a complete transection SCI in rats. Bladder function was followed up over 4 weeks after SCI with subsequent bladder tissue analysis.

The key finding of this pilot study was that montelukast had a positive influence on the preservation of bladder function after SCI. This finding is interesting, as the chosen SCI injury model was a complete transection to exclude any spared neuronal communication to the bladder.

On the functional level, montelukast-treated SCI rats had lower intravesical pressures, less residual volumes, and a better micturition profile. Ultimately, besides the clinical improvement, it was impossible to rule out the presence of DSD in the montelukast-treated SCI animals. Taken together, our results of the urodynamic measurements and clinical assessment indicate preserved sphincteric activity.

As the observation period during this pilot study was limited to 4 weeks post-SCI, it is somewhat early to speculate about the treatment success progression for a longer chronic follow-up. Furthermore, whether or not fibrotic changes, which follow the initial functional deficits, are also slowed down or diminished remains to be clarified in the future.

Even though the CNS is known to be an immune-privileged site, an SCI induces a transient systemic immune-depression with an array of orchestrated molecular and cellular actions in the context of neuroinflammation [7]. There is still an ongoing debate on the positive versus detrimental roles of neuroinflammation, but anti-inflammatory approaches have shown some efficacy in improving functions in SCI animal models [26]. It is unknown which specific aspects of the huge spectrum of neuroinflammatory responses need to be addressed in order to exploit its full potential. Furthermore, harnessing the inflammatory response could be more beneficial than eliminating it. In this explorative study, we focused on eliminating the cysteinyl leukotrienes during the initial SCI phase. Previous studies have shown that the levels of leukotrienes are elevated after SCI in rodent and canine models and in humans [27,28,29]. Montelukast is a well-established and long-term FDA-approved anti-asthmatic drug, which is known to have several beneficial effects on the urinary bladder. Oral montelukast treatment significantly decreased urinary frequency and pain in patients with interstitial cystitis [23]. Even more relevant for this study was the finding that a single intraperitoneal injection of montelukast maintained bladder tissue integrity after a contusion SCI in rats after only one week of SCI [25].

Here, we could confirm that the above-described short-term findings are achievable in a more clinical setting with oral administration of a clinically approved dosage of montelukast over a longer period of time post-SCI.

To compare the pharmacokinetics of montelukast in the healthy Lewis rat strain with humans and other animal species, plasma levels were monitored over time. While plasma concentrations in healthy rats were comparable to those reported in mice, rats, and humans [30,31], they were significantly lower following spinal cord injury (SCI), though still considered relevant to exert a therapeutic effect. We hypothesize that, after initial hepatic first-pass metabolism, a substantial amount of montelukast is distributed into tissues such as the spinal cord and other peripheral tissues affected by SCI, for example, the bladder. Additionally, we observed a significant increase in montelukast levels in cerebrospinal fluid (CSF) over four weeks post-SCI, which may indicate a compromised blood-spinal cord barrier. In healthy control rats and humans, CSF concentrations are similar (3.6 ng/mL in humans versus 2.7 ng/mL in rats), but after SCI, rat CSF levels were six times higher (ranging around 18 ng/mL). Consequently, montelukast mitigates neuroinflammation in the spinal cord [29], which could beneficially influence peripheral organ function. However, due to the complete transection of the spinal cord in our explorative study, we did not observe similar outcomes at the site of injury.

This pilot study highlights the feasibility of chronic montelukast treatment for nLUTD after SCI and shows promising preservation of LUT function over four weeks. While anti-inflammatory effects were only preliminarily assessed via leukotriene levels, these results support further research. Given the central role of inflammation in SCI pathology, future studies should better explore montelukast’s mechanism and its relevance for bladder-targeted therapies. Ideally, this should be done during longer follow-up post-SCI to cover all stages of neuroinflammation and resolution, as well as a further focus on therapy effects within the spinal cord (e.g., on astrogliosis). Furthermore, an analysis of the effects of MLK on the non-damaged neuronal tissue could help to determine the full effect of this treatment in the future.

## 4. Materials and Methods

### 4.1. Animals

We confirm that all methods were performed in accordance with the relevant Austrian Governmental ethical guidelines (Bundesministerium für Bildung, Wissenschaft und Forschung; TVG2012) for animal experiments. This study was carried out under the protocols approved by the Austrian Governmental Ethics Committee for Animal Research with the research numbers BMWFW-66.019/0009-WF/V/3b/2016 and BMWFW-66.019/0024-WF/V/3b/2017. We certify that the common ARRIVE guidelines and all applicable institutional and governmental regulations, as well as local guidelines concerning the ethical use of animals, were followed. Fifty female, 3-month-old strain Lewis rats from Charles River Laboratories (Sulzfeld, Germany) were used for this study. Upon arrival, all animals underwent two weeks of accommodation and handling. Animals were randomly subjected to one of the following four groups: healthy control group without montelukast treatment (CTRL-MLK; n = 5), control with montelukast treatment (CTRL + MLK; n = 5), SCI group without montelukast treatment (SCI-MLK; n = 20), SCI group with montelukast treatment (SCI + MLK; n = 20). The character of the study was purely explorative; therefore, no blinding or sham-SCI groups were included.

### 4.2. Permanent Bladder Catheter Implantation

For a detailed implantation protocol, please refer to the following publications [4,32]. In general anesthesia (mixture of medetomidine (0.15 mg/kg), midazolam (0.08 mg/kg), and fentanyl (0.01 mg/kg), intramuscularly), a PE-50 bladder catheter was implanted via the bladder dome into the bladder. The subcutaneously tunneled bladder catheter was exteriorized at the level of the scapulae and fixed to an infusion harness.

Animals remained in these harnesses throughout the course of the experiment. For analgetic and antibiotic coverage, Meloxicam (2 mg/kg, 2x daily, s.c.) and Baytril (5 mg/kg, 1x, s.c.) were administered for the first five post-surgical days. Antibiotics were given at regular intervals throughout the whole follow-up period to avoid ascending infections due to the permanent catheter implant. During post-SCI follow-up care, urinary bladders were manually expressed twice daily, the expressed urine was caught, and volumes were noted.

### 4.3. Spinal Cord Transection

In general anesthesia (mixture as described above), a standard laminectomy was done at thoracic segmental level T8/T9, and the spinal cord was completely transected and optically verified. A piece of gel foam sponge was used to suppress bleeding after SC transection and left in place. The skeletal muscle layers were thereafter sutured up in layers, and the skin was closed. Post-surgical analgesics and antibiotics were administered as described above for the first seven subsequent post-SCI days. Animals were closely monitored once daily for general health, including weight, fur, eyes, activity level, and stool. Urinary bladder and locomotion were scored separately.

The spinal cord tissue of all injured animals was finally evaluated to reconfirm the completeness of the spinal cord transection.

### 4.4. Montelukast Administration

Montelukast (FDA, Singulair ^®^, application number 20–829) was given orally in a standard dose of 10 mg/kg body weight (dissolved in a methylcellulose gel (KH2PO4; 0.01 M; pH 6.5). Treatment started on day one post-SCI and continued once daily for the whole 28-day follow-up period.

### 4.5. Awake Cystometric Analyses

For analysis, awake animals were placed in a restrainer. After connecting the catheter to the syringe pump, pre-warmed saline solution (Fresenius Kabi, Graz, Austria) was infused into the bladder at a constant rate of 120 µL/min. Infusion was maintained until a minimum of three micturition cycles were measured, but for at least 45 min when no micturition event occurred. Main read-out parameters were the minimal intravesical pressure, threshold pressure, maximum intravesical pressure, average pressure, voided volume, voiding time, and average flow. For data assessment, all pressure and voiding parameters were analyzed at baseline and post-injury at dpi 1, 7, 14, 21, and 28.

### 4.6. Locomotor Scoring

Rats were allowed to move freely and were scored by two blinded experimenters for their ability to use the hind limbs according to the 21-point BBB locomotion scale [33]. Rat joint movement, paw placement, weight support, stepping, forelimb/hind limb coordination, trunk position/stability, and tail position were analyzed at baseline and post-SCI at dpi 1, 15, and 29.

### 4.7. Fluid Analyses

Whole blood samples were taken at one, three, and seven hours, and CSF was additionally harvested at seven hours after montelukast administration to monitor montelukast plasma levels in healthy animals. Furthermore, whole blood samples and CSF were taken once in general anesthesia, three hours after montelukast administration prior to euthanasia at day 29 of follow-up of our SCI collective. Whole blood samples were stored at 4 °C until centrifugation at 1 rcf at 4 °C for 10 min. Plasma was harvested and stored at −20 °C until liquid chromatography-mass spectrometry (LC-MS/MS) analysis. CSF samples were stored at −80 °C directly after sampling until analysis.

The LC-MS/MS method for the quantification of montelukast (Santa Cruz Biotechnology, Santa Cruz, TX, USA) in plasma was described earlier [34] with a modified sample preparation as used in [35]. Briefly, sample preparation consisted of a generic protein precipitation protocol. Therefore, 12.5 µL 100% FA was added to 50 µL of plasma and vortexed briefly before the addition of 150 µL 100% ACN containing montelukast-d6 (Alsachim, France) as internal standard. CSF samples were pooled (unless otherwise indicated) and 100% FA as well as ACN containing montelukast-d6 were added in a ratio of 1:4 (vol/vol) and 1:3 (vol/vol), respectively. After vortexing for two minutes, all samples were centrifuged at 10,500× *g* for 10 min at 4 °C. For plasma samples, 20 µL of the clear supernatant was transferred to 40 µL of mobile phase A. The supernatants of CSF extractions were dried under a constant flow of nitrogen at 45 °C. Completely dried CSF samples were reconstituted in 50 µL 1 mM ammonium formate in 25/75 (vol/vol) acetonitrile/water containing 0.1% FA. Chromatographic separation was carried out with an Agilent 1200 series quaternary HPLC system using a Chromolith Performance RP18-e column (100 × 3 mm, Sorbent Lot/Column No. U12015/039) from Merck (Darmstadt, Germany), operated at a temperature of 40 °C at a flow rate of 0.5 mL/min. We used 1 mM ammonium formate in water containing 0.1% FA as mobile phase A and 1 mM ammonium formate in 95/5 (vol/vol) acetonitrile/water containing 0.1% FA as mobile phase B. Gradient elution started from 25% to 95% B in 10.0 min, followed by a flushing step with 95% B for 0.8 min and re-equilibration with 25% B for 3.2 min. Total time for a single chromatographic run was 14.0 min. Injection volumes were 20 µL for plasma and 30 µL for CSF samples. Selected reaction monitoring (SRM) for montelukast as well as for the d6-internal standard in obtained samples was performed on an API 4000 LC-MS/MS triple quadrupole system (AbSciex) in positive ionization mode. Source temperature was 600 °C, with collision gas of 7 (AU), curtain gas of 25 (AU), ion source gas 1 of 60 (AU), ion source gas 2 of 50 (AU), ion spray voltage of 5500 V, and entrance potential of 10 V. The quantifier ion transitions of MS/MS detection were m/z 586.2→568.2 for montelukast and m/z 592.2→574.4 for montelukast-d6. Calibration levels for plasma samples were prepared in the concentration range of 0.2–2000 ng/mL for plasma and 0.5–50 ng/mL for CSF. Calibration curves were derived from ratios of the peak areas of montelukast and the internal standard, which were plotted against exact concentrations of working standard solutions added to blank plasma or blank CSF. LOD and LOQ were 0.2 ng/mL and 0.6 ng/mL for plasma, and 0.4 ng/mL and 1.2 ng/mL for CSF, respectively, as determined by manually generating extracted ion chromatograms (XICs; n = 5). The intraday and inter-day coefficients of variation (n = 6; 50 ng/mL) for plasma montelukast were 5.1% and 8.5%, respectively. Analyst software 1.6.2 (last accessed 28 August 2024) was used for detection, analysis, and quantification of data.

### 4.8. Euthanasia and Histology

At day 29 of follow-up post-SCI, animals were deeply IV anaesthetized (mixture of Ketamine (273 mg/kg), Xylazine (7.1 mg/kg) and Acepromazine (0.625 mg/kg)) and transcardially flushed with ice cold heparinized saline (0.9% NaCl with 10 units/mL Heparin) until no blood discard was observable, followed by perfusion fixation with ice cold 4% paraformaldehyde (PFA) dissolved in 0.1 M PO4 buffer. After perfusion, a 1 × 1 cm large piece of the lateral bladder wall was dissected and post-fixed for 2 h in 4% PFA before being thoroughly washed and stored in phosphate-buffered saline (PBS) with 0.05% sodium azide at 4 °C until further processing. The spinal cords were harvested and analyzed to confirm the completeness of spinal cord transection.

Bladder specimens were embedded in paraffin via an increasing ethanol series (70% to 100%), methylbenzoat and butylacetat (all chemicals Carl Roth, Karlsruhe, Germany), and serially sectioned at 10 mm. For histological examinations of the smooth muscle and elastic fibers, an Orcein staining was done following initial deparaffinization. The stained sections were photographically documented on a Slide Scanner (Olympus VS120, Olympus Europa SE & Co. KG, Hamburg, Germany), followed by qualitative analysis. For immunohistochemical analyses, sections were de-paraffinized with a prior antigen retrieval with 10 mM sodium citrate buffer in a hot steam bath for 15 min. Primary antibody (Anti-Uroplakin III, ab78196, Abcam, Cambridge, UK, diluted 1:100) was incubated overnight at 4 °C. After washing, secondary antibody (Donkey anti-Mouse Alexa Fluor 568, Thermo Fisher, Dreieich, Germany, diluted 1:000) was incubated for 1 h at room temperature. Nucleic staining (DAPI, 5 min at room temperature) was done as final step. The stained sections were photographically documented on the Slide Scanner VS120 (Olympus, Hamburg, Germany).

### 4.9. Statistics

Experimental data were processed by a spreadsheet and statistics tool (GraphPad Prism Version 10.3.0., La Jolla, CA, USA) to compute mean values, standard deviations, and statistical significance. Data was analyzed by the unpaired *t*-test with Welch correction using a two-stage step-up method with False Discovery Rate. *p* values ≤ 0.05, ≤ 0.01, and ≤ 0.001 were considered significant and marked in the artwork accordingly.

## Figures and Tables

**Table 1:** Comparative analysis of all urodynamic parameters of montelukast-treated healthy control animals (CTRL) and spinal cord injured animals (SCI). Statistical test: unpaired *t*-test, two-tailed, *p*-value for significance: *p* < 0.05.

Urodynamic Parameter	CTRL, Treated	SCI, Treated	*p*-Value
Maximum intravesical pressure (cmH_2_O)	36.60 ± 7.99	35.07 ± 17.55	0.745
Minimum intravesical pressure (cmH_2_O)	4.82 ± 2.45	4.76 ± 2.60	0.937
Threshold intravesical pressure (cmH_2_O)	12.26 ± 2.24	11.25 ± 7.78	0.622
Average intravesical pressure (cmH_2_O)	11.93 ± 4.40	13.70 ± 7.59	0.388
Voided volume (ml)	0.50 ± 0.32	0.34 ± 0.32	0.099
Voiding time (s)	4.43 ± 2.48	7.62 ± 6.52	0.156
Average flow (µL/s)	109.90 ± 64.36	72.06 ± 83.03	0.102

## Data Availability

The datasets used during the current study are available from the corresponding author on reasonable request.
